# Breeding latitude predicts timing but not rate of spring migration in a widespread migratory bird in South America

**DOI:** 10.1002/ece3.5159

**Published:** 2019-04-16

**Authors:** Alex E. Jahn, Joaquín Cereghetti, Víctor R. Cueto, Michael T. Hallworth, Douglas J. Levey, Miguel Â. Marini, Diego Masson, Marco A. Pizo, José Hernán Sarasola, Diego T. Tuero

**Affiliations:** ^1^ Departamento de Zoologia Universidade Estadual Paulista Rio Claro Brazil; ^2^ Facultad de Ciencias Exactas y Naturales Universidad Nacional de La Pampa La Pampa Argentina; ^3^ Centro de Investigación Esquel de Montaña y Estepa Patagónica (CIEMEP) Consejo Nacional de Investigaciones Científicas y Técnicas (CONICET) y Universidad Nacional de la Patagonia ‘‘San Juan Bosco’’ Esquel Argentina; ^4^ Migratory Bird Center Smithsonian Conservation Biology Institute Washington, DC USA; ^5^ National Science Foundation Alexandria Virginia USA; ^6^ Departamento de Zoologia Universidade de Brasília Brasília Brazil; ^7^ Facultad de Ciencias Naturales y Museo Universidad Nacional de La Plata La Plata Argentina; ^8^ Centro para el Estudio y Conservación de las Aves Rapaces en Argentina (CECARA) Universidad Nacional de La Pampa (UNLPam), and Instituto de las Ciencias de la Tierra y Ambientales de La Pampa (INCITAP), Consejo Nacional de Investigaciones Científicas y Técnicas (CONICET) Santa Rosa, La Pampa Argentina; ^9^ Departamento de Ecología, Genética y Evolución, Facultad de Ciencias Exactas y Naturales Instituto IEGEBA (CONICET‐UBA) Universidad de Buenos Aires Buenos Aires Argentina

**Keywords:** Argentina, Brazil, cerrado, life history, light‐level geolocator, Pampas

## Abstract

1. Identifying the processes that determine avian migratory strategies in different environmental contexts is imperative to understanding the constraints to survival and reproduction faced by migratory birds across the planet.
2. We compared the spring migration strategies of Fork‐tailed Flycatchers (*Tyrannus s. savana*) that breed at south‐temperate latitudes (i.e., austral migrants) vs. tropical latitudes (i.e., intratropical migrants) in South America. We hypothesized that austral migrant flycatchers are more time‐selected than intratropical migrants during spring migration. As such, we predicted that austral migrants, which migrate further than intratropical migrants, will migrate at a faster rate and that the rate of migration for austral migrants will be positively correlated with the onset of spring migration.
3. We attached light‐level geolocators to Fork‐tailed Flycatchers at two tropical breeding sites in Brazil and at two south‐temperate breeding sites in Argentina and tracked their movements until the following breeding season.
4. Of 286 geolocators that were deployed, 37 were recovered ~1 year later, of which 28 provided useable data. Rate of spring migration did not differ significantly between the two groups, and only at one site was there a significantly positive relationship between date of initiation of spring migration and arrival date.
5. This represents the first comparison of individual migratory strategies among conspecific passerines breeding at tropical vs. temperate latitudes and suggests that austral migrant Fork‐tailed Flycatchers in South America are not more time‐selected on spring migration than intratropical migrant conspecifics. Low sample sizes could have diminished our power to detect differences (e.g., between sexes), such that further research into the mechanisms underpinning migratory strategies in this poorly understood system is necessary.

## 1 INTRODUCTION

Bird migration across the New World is ubiquitous, from high Arctic tundra to Patagonia, and research on the mechanisms underpinning avian migratory patterns in the New World and beyond continues to be a rapidly growing area of inquiry. Such studies have shown that the timing and pace of migration and the location of migratory routes can vary widely not only between species, but also between populations within a species (e.g., *Sylvia* warblers, Fransson, 1995; Collared flycatchers, *Ficedula albicollis*, Briedis et al., 2016; Northern wheatears, *Oenanthe oenanthe*, Bairlein et al., 2012; Wood thrushes, *Hylocichla mustelina*, Stanley, MacPherson, Fraser, McKinnon, & Stutchbury, 2012; White‐crested elaenias, *Elaenia albiceps*, Bravo, Cueto, & Gorosito, 2017). Understanding the causes of such variation is vital for developing a basic understanding of the evolution and regulation of migration, as well as for evaluating the fitness consequences of employing a given migratory strategy, since processes that occur in one season can influence the survival and reproduction of an individual in subsequent seasons (i.e., through carryover effects, reviewed by Harrison, Blount, Inger, Norris, & Bearhop, 2011).

Migratory birds that initiate reproduction late due to delayed arrival at the breeding site risk lower reproductive success, due to a steep decline in number of young fledged as the breeding season progresses (e.g., Murphy, 1986; Verboven & Visser, 1998). For example, in Hoopoes (*Upupa epops*), the duration of spring migration is negatively related to breeding territory quality and number of fledglings produced (van Wijk, Schaub, & Bauer, 2017). In contrast, an earlier arrival at the breeding site can result in increased reproductive success by increasing the probability of successfully raising a brood of nestlings or by allowing an individual to attempt multiple broods (Smith & Moore, 2005). Because spring migration strategy and timing of arrival on breeding grounds can have profound consequences on a migrant's ability to successfully reproduce in a given year (Kokko, 1999; McKinnon, Stanley, & Stutchbury, 2015; Smith & Moore, 2005; Visser, Holleman, & Gienapp, 2006), arrival timing is presumably under strong selection (Smith & Moore, 2005), especially in males, which must often compete for breeding territories and mates (Morbey & Ydenberg, 2001; Tøttrup & Thorup, 2008). Under optimal bird migration theory, such birds are thought to be time‐selected migrants (Åkesson & Hedenström, 2007; Alerstam & Lindström, 1990; Hedenström, 2008).

Despite the importance of migratory timing on reproductive success, we lack a detailed understanding of the selective pressures molding bird migration strategies across taxa and, except for a handful of model species, between conspecific populations. Hundreds of migratory bird species spend their entire lives within tropical and south‐temperate latitudes, and their migratory strategies are poorly known. Intratropical migratory birds breed, migrate, and overwinter entirely within the tropics (including Mesoamerica and the Caribbean, South America, Africa, and Asia). Austral migrants breed at southern temperate latitudes of Africa, South America, and Australia and overwinter closer to the Equator (reviewed by Chesser (1994), Dingle (2008), Faaborg et al. (2010)). Research on these poorly understood systems promises novel insights into how much variation in migratory strategies exists across regions and taxa, and ultimately the mechanisms that underpin the strategies employed in these different systems.

Two lines of evidence suggest that intratropical migrants should employ a different spring migration strategy than austral migrants that breed at south‐temperate latitudes. First, birds that breed at tropical latitudes experience a different ecological context than those that breed at temperate latitudes. Compared to seasonality at temperate latitudes, seasons in the tropics are primarily defined by variation in rainfall (e.g., Gottsberger & Silberbauer‐Gottsberger, 2006; Oliveira & Marquis, 2002; Wikelski, Hau, & Wingfield, 2000), which in turn drives timing of leafing, flowering, and fruiting of tropical plants (e.g., Araujo, Vieira‐Filho, Barbosa, Diniz‐Filho, & Silva, 2017; Myneni et al., 2007; Patrícia et al., 2000), and consequently the abundance of arthropods (Amorim, DeÁvila, Camargo, Vieira, & Oliveira, 2009; Cotton, 2007; Develey & Peres, 2000; Jahn et al., 2010; Pinheiro, Diniz, Coelho, & Bandeira, 2002). Such seasonality in precipitation is more unpredictable between years than is temperature (Lisovski, Ramenofsky, & Wingfield, 2017). In the Southern Hemisphere, the degree of seasonality (i.e., predictability and amplitude) peaks at south‐temperate latitudes (i.e., at ~35°S; Lisovski et al., 2017), such that austral migrants experience more predictable seasonality than intratropical migrants. Second, optimal migration theory postulates that birds that migrate longer distances should be more time‐selected on migration (Alerstam & Lindström, 1990; Hedenström, 2008). Thus, because austral migrants that overwinter in the tropics and breed at south‐temperate latitudes breed at seasonally more predictable sites and migrate longer distances than intratropical migrants, austral migrants should be more time‐selected on spring migration than intratropical migrants.

Our objective was to compare migratory strategies of birds that migrate different distances in South America. To do so, we deployed light‐level geolocators on migratory Fork‐tailed Flycatchers (*Tyrannus s. savana*) in South America. This subspecies breeds from central Brazil to central Argentina (Mobley, 2004), with Brazilian populations breeding at tropical latitudes and overwintering in northern South America (Jahn, Giraldo, et al., 2016; Jahn, Seavy, et al., 2016); they are thus intratropical migrants. Populations that breed at south‐temperate latitudes in Argentina also overwinter in northern South America (Jahn et al., 2013) and are thus Neotropical austral migrants (Cueto & Jahn, 2008). Fall migration patterns (Jahn, Giraldo, et al., 2016; Jahn, Seavy, et al., 2016; Jahn et al., 2013), tracking of environmental conditions (MacPherson et al., 2018), and, for one site in Brazil, timing of arrival of males versus females (Bejarano & Jahn, 2018) have been studied in Fork‐tailed Flycatchers, but little is known about spring migration strategies.

Because Fork‐tailed Flycatchers that breed at south‐temperate latitudes (hereafter, “austral migrants”) migrate a longer distance in spring than those that breed at tropical latitudes (hereafter, “intratropical migrants”), we hypothesize that austral migrants will employ a more time‐selected spring migration strategy than intratropical migrants. We predict that, compared to intratropical migrants, austral migrants on spring migration should exhibit: (a) an overall greater migration rate and (b) a spring migration rate that is positively related to the date of departure on spring migration. We also evaluate stopover duration of intratropical versus austral migrants. Optimal migration theory predicts that time‐selected migrants should minimize time spent on stopover when fuel deposition rates are high (Alerstam & Hedenström, 1998; Lindström & Alerstam, 1992), continuing to migrate upon reaching optimal fuel load (Alerstam & Lindström, 1990). We make no predictions regarding stopover duration of Fork‐tailed Flycatchers, since we have no information on their refueling rates during stopover.

## 2 MATERIALS AND METHODS

**Figure 1:**
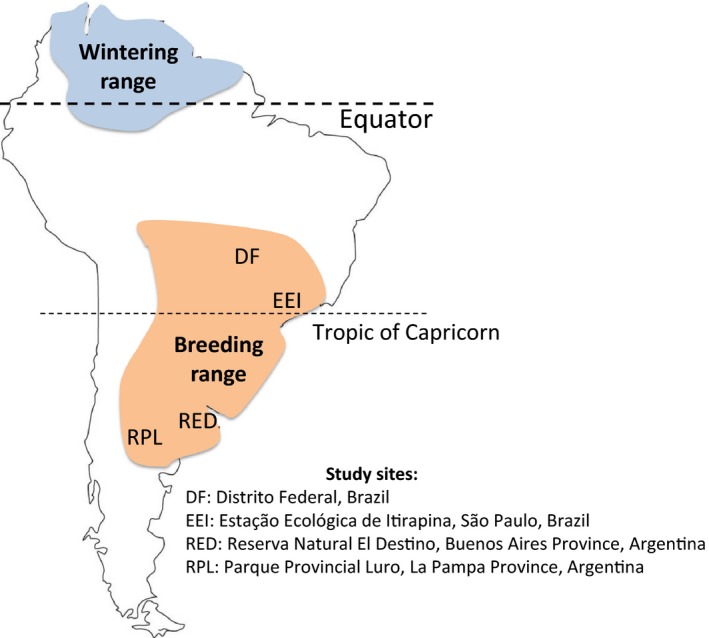
Location of study sites and range of the Fork‐tailed Flycatcher in South America

We captured flycatchers during the breeding season (September to December in Brazil; Marini, Lobo, Lopes, Franca, & Paiva, 2009; October to January in Argentina; Jahn et al., 2014), at four sites: (a) Parque da Alvorada (an urban park) and the campus of the Universidade de Brasília, Distrito Federal, Brazil (hereafter, “DF”; 15.8°S, 47.8°W) in the city of Brasília, where habitat is primarily composed of mowed grass with scattered trees (Figure 1). We captured flycatchers there in October and November 2013, September and October 2014, and October 2015. (b) Estação Ecológica de Itirapina, São Paulo State, Brazil (“EEI”; 22.3°S, 47.9°W), which is primarily composed of low campo and cerrado grassland (Figure 1). We captured flycatchers there in November 2013, September to December 2014, and October 2015. (c) Reserva Natural El Destino, Buenos Aires Province, Argentina (“RED”; 35.1°S, 57.4°W), where habitat is primarily temperate grasslands and marshes, intersected by woodland‐dominated *Celtis ehrenbergiana* and *Scutia buxifolia* (Figure 1). We captured flycatchers there from December 2009 to February 2010, December 2010 to January 2011, October 2011 to January 2012, November 2012 to January 2013, December 2013 to January 2014, November 2014 to January 2015, and December 2015 to January 2016. (d) Reserva Provincial Parque Luro, neighboring private properties, and on a nearby road right‐of‐way (“RPL”; 36.8°S, 64.3°W) in La Pampa Province, Argentina (Figure 1). Habitat there consists of tracts of *Prosopis caldenia* woodland and grassland matrix, with nearby agricultural fields. We captured flycatchers there in December 2013, and November 2014 to January 2015.

At all sites, flycatchers were captured by placing a predator model (e.g., Chimango Caracara, *Milvago chimango*; Southern Crested Caracara, *Caracara plancus*) or a speaker emitting a conspecific call two meters or less from one or two 3 × 12 or 3 × 18 m polyester or nylon mist nets (38 mm mesh size). Nets were placed 2–4 m from a flycatcher nest that contained either eggs or nestlings. Upon capture, flycatchers were banded with an individually numbered metal band and up to three Darvic color bands. Flycatchers were aged and sexed using the shape of the notch of primaries 8–10 and/or the presence of a brood patch or cloacal protuberance (Jahn, Giraldo, et al., 2016; Jahn, Seavy, et al., 2016; Pyle, 1997). Wing chord, tarsus length, and tail length were collected following methods in Ralph, Guepel, Pyle, Martin, and DeSante (1993), and body mass was measured to the nearest 0.1 g using a portable digital scale (Ohaus LS 200). Finally, flycatchers were outfitted with a light‐level geolocator using a leg‐loop harness (Rappole & Tipton, 1991) made of Filament Kevlar (500 tex; Saunders Thread, Gastonia, North Carolina, USA). Geolocator model type varied during the study: During the 2009 breeding season, we used model Mk10S geolocators (1.2 g; British Antarctic Survey, Cambridge, UK), during the 2010 breeding season, we used model Mk12S geolocators (0.9 g; British Antarctic Survey, Cambridge, UK), and during the 2013 and 2014 breeding seasons, we used model P65C2‐11 geolocators (0.8 g; MigrateTech, Inc., Cambridge, UK). The mean mass of the flycatchers on which we deployed geolocators was 31.0 g (±2.19 g *SD*), and the mean relative mass of the geolocators to flycatchers was 2.7% (±0.37). In no case did the combined mass of the harness and geolocator exceed 4% of the mass of the flycatchers on which they were deployed.

We deployed 55 geolocators at DF and 58 geolocators at EEI (Brazil). We recovered 4 (7%) geolocators at DF, and recovered 8 (14%) at EEI, all of which had usable data (two geolocators had been deployed on the same individual in different seasons at EEI, see explanation below). We deployed 103 geolocators at RED and 70 geolocators at RPL (Argentina). We recovered 16 (16%) at RED, of which nine had usable data, and we recovered 9 (13%) at RPL, of which eight had usable data. This rate of geolocator recovery is within the range of that reported in studies of other migratory birds (Bridge et al., 2013). None of the recaptured flycatchers exhibited any sign of injury from the geolocator or harness. We were not able to recapture many flycatchers because they had become net shy and much less responsive to predator models and playbacks, especially in Brazil. Overall, we analyzed data from one female and 10 male intratropical migrant flycatchers from Brazil, and six female and 11 male austral migrant flycatchers from Argentina (Table 1).

**Table 1:** Individual migration histories of Fork‐tailed Flycatchers breeding at south‐temperate latitudes (i.e., austral migrants) and tropical latitudes (i.e., intratropical migrants) in South America. All durations are expressed in days and distances in km, and spring migration rate in km/day

ID	Site	Sex	No. of winter sites	Winter duration	No. of spring stopover sites	Spring duration	Spring distance	Spring rate	Spring stopover duration	Spring initiation	Spring arrival	
Austral migrant flycatchers (Argentina)												
1	RED/Buenos Aires	F	2	169	5	45	4,353	96.7	38	29‐Aug‐11	12‐Oct‐11	
2	RED/Buenos Aires	F	1	133	0	18	2,922	162.3	0	23‐Sep‐10	10‐Oct‐10	
3	RED/Buenos Aires	F	2	183	1	NA	4,816	NA	5	27‐Sep‐14	NA	
4	RED/Buenos Aires	F	3	177	3	27	4,555	168.7	18	21‐Sep‐15	17‐Oct‐15	
5	RED/Buenos Aires	M	2	182	4	43	3,979	92.5	23	30‐Aug‐11	11‐Oct‐11	
6	RED/Buenos Aires	M	2	137	5	51	4,705	92.3	42	13‐Aug‐11	2‐Oct‐11	
7	RED/Buenos Aires	M	2	126	3	36	4,259	118.3	30	8‐Sep‐10	13‐Oct‐10	
8	RED/Buenos Aires	M	2	185	3	32	4,618	144.3	11	9‐Sep‐14	10‐Oct‐14	
9	RED/Buenos Aires	M	2	142	4	21	4,682	223.0	15	15‐Sep‐14	5‐Oct‐14	
10	RPL/La Pampa	F	4	155	2	31	4,926	158.9	25	5‐Sep‐14	5‐Oct‐14	
11	RPL/La Pampa	F	2	131	2	57	4,644	81.5	47	29‐Aug‐14	24‐Oct‐14	
12	RPL/La Pampa	M	3	152	4	29	4,896	168.8	17	12‐Sep‐14	10‐Oct‐14	
13	RPL/La Pampa	M	1	158	3	40	4,383	109.6	28	30‐Aug‐14	8‐Oct‐14	
14	RPL/La Pampa	M	5	151	1	23	3,311	143.9	2	29‐Sep‐14	21‐Oct‐14	
15	RPL/La Pampa	M	2	147	2	47	4,351	92.6	29	30‐Aug‐14	15‐Oct‐14	
16	RPL/La Pampa	M	1	128	2	29	4,918	169.6	21	7‐Sep‐14	5‐Oct‐14	
17	RPL/La Pampa	M	2	172	4	41	5,136	125.3	24	29‐Aug‐14	8‐Oct‐14	
		Mean	2.2	154.6	2.8	35.6	4,438.4	134.3	22.1	8‐Sep	11‐Oct	
		*SD*	1.03	20.31	1.42	11.24	578.62	39.55	13.30			
Intratropical migrant flycatchers (Brazil)												
18	DF/Brasília	M	1	88	2	25	2,844	113.7	13	18‐Jul‐14	11‐Aug‐14	
19	DF/Brasília	M	1	111	1	16	2,604	162.8	8	4‐Aug‐14	19‐Aug‐14	
20	DF/Brasília	M	1	114	3	20	3,290	164.5	15	22‐Jul‐14	10‐Aug‐14	
21	DF/Brasília	M	1	146	3	20	3,259	162.9	10	2‐Aug‐14	21‐Aug‐14	
22	EEI/São Paulo	F	1	139	3	25	3,037	121.5	16	12‐Aug‐15	5‐Sep‐15	
23	EEI/São Paulo	M	1	145	3	29	3,757	129.6	10	16‐Aug‐14	13‐Sep‐14	
24	EEI/São Paulo	M	3	169	1	22	3,587	163.1	8	12‐Aug‐14	2‐Sep‐14	
25	EEI/São Paulo	M	1	158	3	32	3,818	119.3	15	5‐Aug‐14	5‐Sep‐14	
26	EEI/São Paulo	M	3	155	4	29	3,523	121.5	24	31‐Jul‐14	28‐Aug‐14	
27	EEI/São Paulo	M	3	161	2	19	3,628	190.9	9	16‐Aug‐14	3‐Sep‐14	
28	EEI/São Paulo	M	1	147	4	27	3,555	131.7	17	28‐Jul‐14	23‐Aug‐14	
		Mean	1.5	139.4	2.6	24.0	3,355	143.8	13.2	4‐Aug	26‐Aug	
		*SD*	0.93	24.82	1.03	5.00	388.90	25.69	4.87			

Note

DF: Distrito Federal, Brazil; EEI: Estação Ecológica de Itirapina, São Paulo State, Brazil; RED: Reserva Natural El Destino, Buenos Aires Province, Argentina; RPL: Reserva Provincial Parque Luro and neighboring private properties, La Pampa Province, Argentina; *SD*: standard deviation.

### 2.1 Data analysis

Raw light‐level data were transformed into geographic location estimates through the Solar/satellite Geolocation for Animal Tracking package (SGAT; Sumner, Wotherspoon, & Hindell, 2009; Wotherspoon, Sumner, & Lisovski, 2013), which incorporates error inherent in light‐level geolocation. We determined sunrise and sunset times (twilight events) using a light threshold of 0.5, identified using the findTwilights function in the TwGeos package (https://github.com/slisovski/TwGeos). For each Fork‐tailed Flycatcher, we specified a model that included geographic locations derived using the threshold method, a log‐normal model that described the error distribution between estimated and known sunrise and sunset times, a beta‐distributed behavioral model that described potential flight speeds, and a land mask that constrained stationary periods to land. We used light levels recorded by geolocators while individuals were known to be at the capture location to (a) describe the error distribution between estimated and known sunrise and (b) derive zenith angles (angle of the sun with respect to vertical when light data cross a specified threshold). A Metropolis sampler was used to run Markov Chain Monte Carlo (MCMC) simulations. For each individual, we ran the model three times with 5,000 MCMC iterations on three chains per run. We treated the first two runs as a burn‐in period while summarizing location estimates between each run. We used the resulting median daily location to initialize each subsequent run. We kept every second iteration from the posterior distribution, from which we drew our geographic inference.

We delimited the daily geographic estimates provided by light‐level geolocators into a series of stationary periods, using the MigSchedules function in the “LLmig” R package available at https://github.com/MTHallworth/LLmig. The MigSchedules function is similar to the popular ChangeLight function in the GeoLight package (Lisovski & Hahn, 2012) but incorporates location uncertainty inherent in light‐level geolocation (e.g., Lisovski et al., 2018) when determining stationary periods. The MigSchedules function uses natural breaks (changes in the running posterior mean) in the posterior location estimates from the MCMC simulations to determine stationary periods and has an option to use only longitude during the equinox period to estimate movements, which we did not do because stopovers occurred within the same longitude as the breeding site (see below).

**Figure 2:**
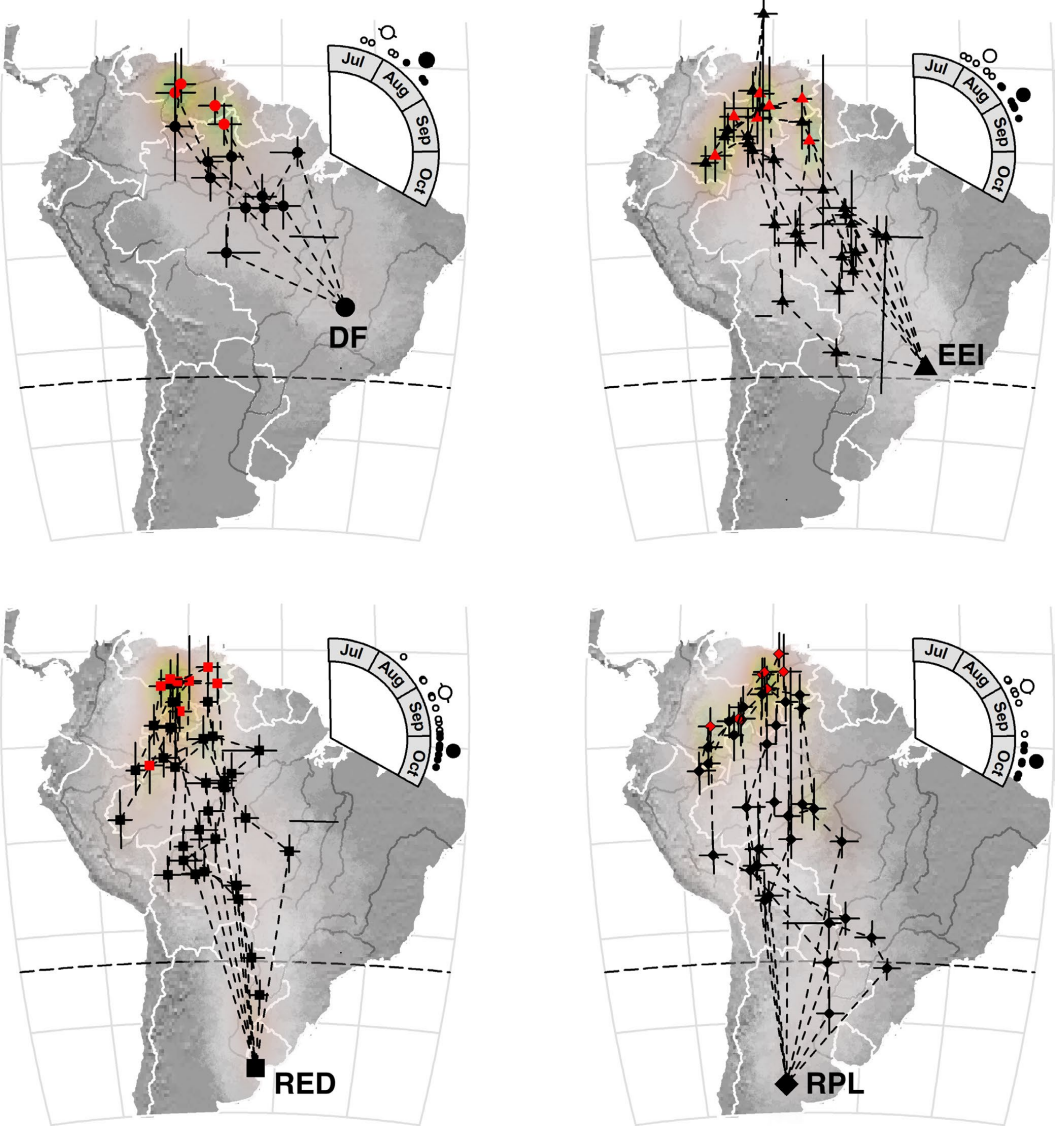
Spring migratory routes and stopover sites of Fork‐tailed Flycatchers from four breeding sites in South America. The large symbols on each map depict the breeding site, small red symbols represent winter stationary periods, and small black symbols represent stopover stationary periods. Lines on polygons represent the 95% credible interval of latitude and longitude. Dotted lines may not represent the actual migratory path taken by individuals between stopover sites. Shading on the northern South American wintering grounds represents location uncertainty. The inset on each map shows the spring departure (small open circles) and subsequent breeding arrival dates (small closed circles) of each individual. The mean departure date (large open circle) and arrival date (large closed circle) for each population are also shown (lines shown on some large circles represent standard error). DF: Distrito Federal, Brazil; EEI: Estação Ecológica de Itirapina, São Paulo State, Brazil; RED: Reserva Natural El Destino, Buenos Aires Province, Argentina; RPL: Reserva Provincial Parque Luro and neighboring private properties, La Pampa Province, Argentina; *SD*: standard deviation

We define the beginning of the winter period as the initiation of first stationary period in fall that was at least 30 days duration. Flycatchers exhibited multiple stationary periods throughout the nonbreeding season (Table 1), often moving across longitudes (Figure 2), such that changes in longitude were not useful to delimit wintering versus migratory periods. Therefore, to estimate the initiation and termination of spring migration, we used large changes in the duration of stationary periods. We define termination of the winter period and initiation of spring migration as the end of the first stationary period that was at least 30 days long and that was not followed by a stationary period of 30 days or more. We define termination of spring migration as the first date the bird was within the 95% credible interval of the breeding site longitude. One breeding site (Reserva Natural El Destino) is located directly south of the spring migration route, such that some flycatchers entered the 95% credible interval of the breeding site longitude while still migrating. As a result, the estimated arrival time at that site using this method gave arrival dates weeks before the first flycatchers were visually detected by us at the site. The arrival date of flycatchers at that site is generally well known since results of transect censuses at that site in early spring by DM show that flycatchers arrive in late September at the earliest, which is during the spring equinox, lending greater uncertainty to our estimates of spring arrival. We therefore modified the definition of arrival in spring at the two study sites in Argentina by restricting arrival to be no earlier than the date that the first flycatcher was observed during censuses conducted in spring at Reserva Natural El Destino during each year of the project (i.e., 2010: 10 October; 2011: 29 September; 2014: 5 October; 2015: 12 October). We also excluded from analysis a flycatcher tagged at Reserva Natural El Destino that migrated ~535 km/day, a rate more than double that of the next fastest individual to that site, and likely a product of our uncertainty in estimates of arrival date. These modifications could bias our results by making the duration of spring migration for flycatchers migrating to Argentina longer, and therefore, their rate of spring migration slower relative to flycatchers captured at study sites in Brazil. However, since we predicted that flycatchers breeding in Argentina should migrate at a faster rate than those breeding in Brazil, they bias our results toward being more conservative.

To estimate spring migration distance, we calculated the great circle distance between the last winter stationary period and the breeding site, using the median latitude and longitude of the last winter stationary period and the latitude and longitude of the breeding site. For flycatchers that had only one winter stationary period, we used the median latitude and longitude of that single winter period to calculate spring great circle migration distance. Great circle distances were calculated using the “sp” and “raster” packages (Hijmans & van Etten, 2014; Pebesma & Bivand, 2005) in program R (R Core Team, 2016).

For one male (#27; Table 1) captured at EEI, we acquired geolocator data across 2 years, since geolocators had been deployed on that individual in two different years (2013 and 2014). We only analyzed geolocator data from this individual for the first year, to avoid violating the assumption of independence of data. We chose to analyze data from the first rather than the second year to make location data from that individual comparable to location data from other individuals, since location data from all other individuals were collected during the first year they carried a tag. We calculated the duration of the overwintering period as the number of days between the last day of the last winter stationary period and the first day of the first winter stationary period. We calculated the duration of spring migration as the number of days between the first day the bird was at the breeding site and the last day of the last winter stationary period. We calculated how much time flycatchers spent on stopover during spring migration as the sum of the duration of all spring stationary periods. We used linear models (LM) in the nlme package in program R v.3.3.3 (R Core Team, 2016) to evaluate the additive and/or interactive effects of breeding site on the duration of spring migration, duration of time spent on stopover, rate of spring migration, spring migration distance, date of departure on spring migration, and date of arrival at the breeding site. We included the additive effect of tarsus length as a proxy for body size (i.e., smaller body size may be selected for under time‐selected migration; Hedenström & Alerstam, 1998), and the interactive effects of wing chord and tail length as fixed factors within each population, since those characters could influence the rate and timing of migration (Hedenström, 2008; Provinciato, Araújo, & Jahn, 2018). We did not include year as a random variable, since we did not obtain migration data from flycatchers in the same years across different sites. We analyzed all variables using a Gaussian error term and an identity link function. We used backward elimination to remove effects that contributed least to the model and compared the goodness of fit of each model using likelihood ratio tests. Finally, we used Pearson's correlation in program R v. 3.3.3 (R Core Team, 2016) to evaluate whether the rate of spring migration and timing of arrival at the breeding site are related to timing of departure on spring migration.

## 3 RESULTS

We found no significant difference between males versus females from Argentinian sites in the rate, distance and duration of spring migration, or in the duration of time spent on stopover, date of departure, or date of arrival at the breeding site (*p* > 0.05 in all cases; Table 1). Thus, we combined data from both sexes for further analyses, except for analysis of variation in arrival date at the breeding site.

### 3.1 General patterns

Intratropical and austral migrants overwintered from western Amazonia (Peru, Colombia, and Brazil) to northeastern South America (Guyana), with most going to the Orinoco River Basin in Colombia and Venezuela (Figure 2). Intratropical migrant flycatchers overwintered at a mean latitude of 2.71°N (±1.51 *SE*) and austral migrants at 2.58°N (±1.06). The winter period lasted from March to August for intratropical migrants and from April to September for austral migrants, lasting an average of ~4.5 months for intratropical migrants and ~5 months for austral migrants (Table 1). Both intratropical and austral migrants used multiple overwintering sites, although most intratropical migrants used only one (Table 1).

Initially, the spring migration route for both intratropical and austral migrants crossed central Amazonia (Figure 2). Intratropical migrants generally arrived soon thereafter at the breeding site, whereas most austral migrants passed through Bolivia or western Brazil and eventually into northern Argentina and Paraguay, before arriving back at the breeding site (Figure 2).

### 3.2 Distance and duration of spring migration

**Figure 3:**
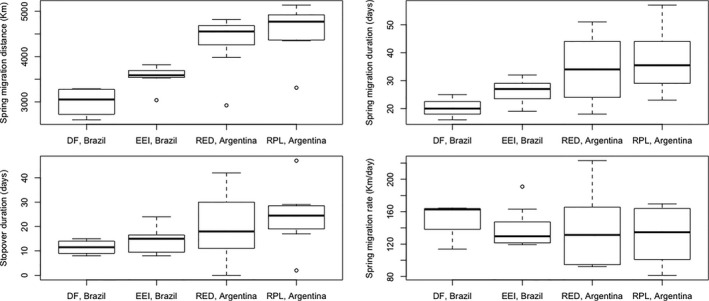
Spring migration distance, spring migration duration, stopover duration, and migration rate of Fork‐tailed Flycatchers as a function of breeding site (i.e., intratropical migrants breeding in Brazil and austral migrants breeding in Argentina). The line inside each box represents the median; the top and bottom of each box represent the upper and lower quartiles, respectively; the lines extending vertically from the top and bottom of each box represent maximum and minimum values, respectively; circles represent outliers

Spring migration distance was significantly different between sites (Table 2), with flycatchers from both sites in Brazil migrating a significantly shorter distance than those from sites in Argentina (Table 2 and Figure 3). However, there was no significant difference in the spring migration distance between flycatchers from sites in Brazil or between those from sites in Argentina (Table 2 and Figure 3). Overall, austral migrants travelled on average ~4,438 km (±578.6) on spring migration at a mean rate of ~134 km/day (±39.6), whereas intratropical migrants migrated on average 3,355 km (±388.9) in spring at a mean rate of 144 km/day (±25.7; Table 1, Figure 3). There was no significant effect of wing chord (LM: *F* = 0.64, *df* = 22, *p* = 0.43), a marginally nonsignificant trend for tarsus length (LM: *F* = 4.23, *df* = 23, *p* = 0.05), and no significant effect of tail length (LM: *F* = 0.00, *df* = 19, *p* = 0.99) in any model.

**Table 2:** Results of linear model output of migration duration, distance, and arrival and initiation dates of spring migration of Fork‐tailed Flycatchers in South America

Variables	Predictor variable	Estimate ± *SE*	*t*	*df*	*p*
Spring duration (days)	Intercept (DF)	20 ± 4	4.32	3, 23	˂*0.01*
	Site: EEI	6 ± 5	1.00		0.32
	Site: RED	13 ± 5	2.42		*0.02*
	Site: RPL	16 ± 5	2.94		˂*0.01*
Spring distance (km)	Intercept (DF)	2,999 ± 246	12.19	3, 24	˂*0.001*
	Site: EEI	558 ± 308	1.81		0.08
	Site: RED	1,321 ± 295	4.47		˂*0.001*
	Site: RPL	1571 ± 301	5.22		˂*0.001*
Spring initiation (Julian date)	Intercept (DF)	208 ± 5	37.19	3, 24	˂*0.001*
	Site: EEI	12 ± 7	1.75		0.09
	Site: RED	44 ± 6	6.56		˂*0.001*
	Site: RPL	40 ± 6	5.97		˂*0.001*
Spring arrival (Julian date)	Intercept (DF)	227 ± 3	73.63	3, 23	˂*0.001*
	Site: EEI	18 ± 3	4.70		˂*0.001*
	Site: RED	55 ± 3	14.75		˂*0.001*
	Site: RPL	57 ± 3	15.28		˂*0.001*

Note

Italics indicate significant results of *p*‐values (*p* < 0.05).

DF: Distrito Federal, Brazil; EEI: Estação Ecológica de Itirapina, São Paulo State, Brazil; RED: Reserva Natural El Destino, Buenos Aires Province, Argentina; RPL: Reserva Provincial Parque Luro and neighboring private properties, La Pampa Province, Argentina; SE: standard error.

The duration of spring migration was also significantly different between sites (Table 2), with flycatchers from RPL, Argentina, spending a significantly longer time on migration than flycatchers from both sites in Brazil (Figure 3). However, there was no significant difference in the duration of migration between flycatchers from the two sites in Brazil or between flycatchers from the two sites in Argentina (Figure 3). Overall, austral migrants spent a mean of ~36 days (±11.2 *SD*) on spring migration and intratropical migrants spent a mean of ~24 days on migration (±5.0; Table 1 and Figure 3). There was no significant effect of wing chord (LM: *F* = 0.07, *df* = 20, *p* = 0.79), tarsus length (LM: *F* = 0.05, *df* = 22, *p* = 0.82), or tail length (LM: *F* = 0.16, *df* = 20, *p* = 0.69) in any model.

### 3.3 Timing and rate of spring migration

Date of departure on spring migration was significantly different between sites, with flycatchers migrating to sites in Argentina departing significantly later than flycatchers migrating to sites in Brazil, and no significant difference in departure date between flycatchers migrating to Brazilian sites or between flycatchers migrating to Argentinian sites (Tables 1 and 2). Likewise, arrival date at the breeding site was significantly different between sites (Tables 1 and 2), with flycatchers from sites in Argentina arriving significantly later than flycatchers migrating to sites in Brazil, and flycatchers arriving at EEI, Brazil, significantly later than at DF, Brazil (Tables 1 and 2). However, there was no significant difference in arrival date between flycatchers migrating to Argentinian sites (Tables 1 and 2). Overall, intratropical migrants initiated spring migration in early August and arrived back at their breeding sites in late August. In contrast, austral migrants initiated spring migration in early September and arrived back at their breeding sites in early October (Table 1). Thus, intratropical migrants initiated spring migration on average 35 days earlier than austral migrants and arrived earlier at the breeding site than austral migrants (on average 45 days earlier; Table 1).

The daily spring migration rate was not significantly different among flycatchers migrating to the four sites (LM: *F* = 1.19, *df* = 3, *p* = 0.338).

### 3.4 Use of stopover sites

Both austral and intratropical migrants used an average of ~3 stopover sites during spring migration (stopover sites are equivalent to stationary periods, see Materials and Methods), and there was no significant difference in the amount of time spent on stopover among flycatchers migrating to the four sites (on average, 22.1 days for austral migrants vs. 13.2 days for intratropical migrants; LM: *F* = 0.468, *df* = 3, *p* = 0.708; Table 1, Figure 3).

### 3.5 Seasonal effects on rate and timing of spring migration

**Figure 4:**
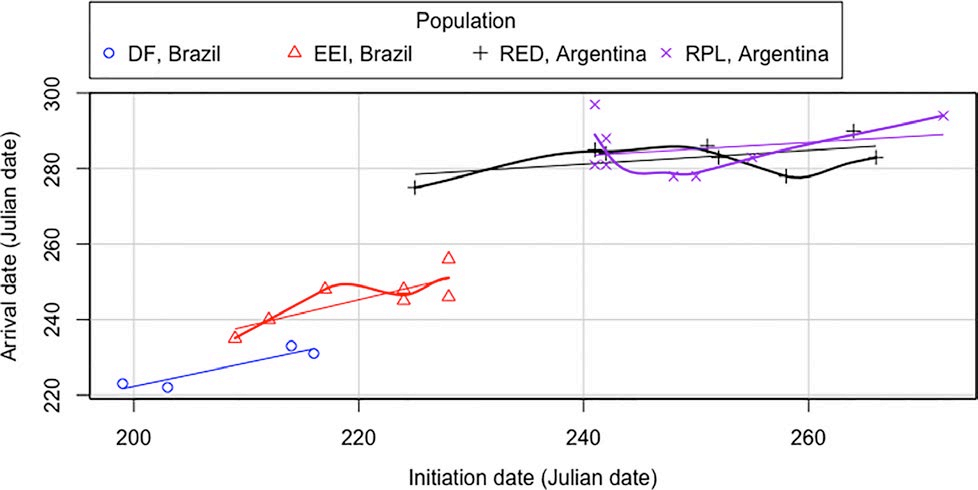
Date of arrival of Fork‐tailed Flycatchers at the breeding site as a function of date of initiation of spring migration (i.e., intratropical migrants breeding in Brazil and austral migrants breeding in Argentina). Straight lines represent the regression line for the population at each site, and curved lines represent the locally weighted smoothing line for the population at each site

**Figure 5:**
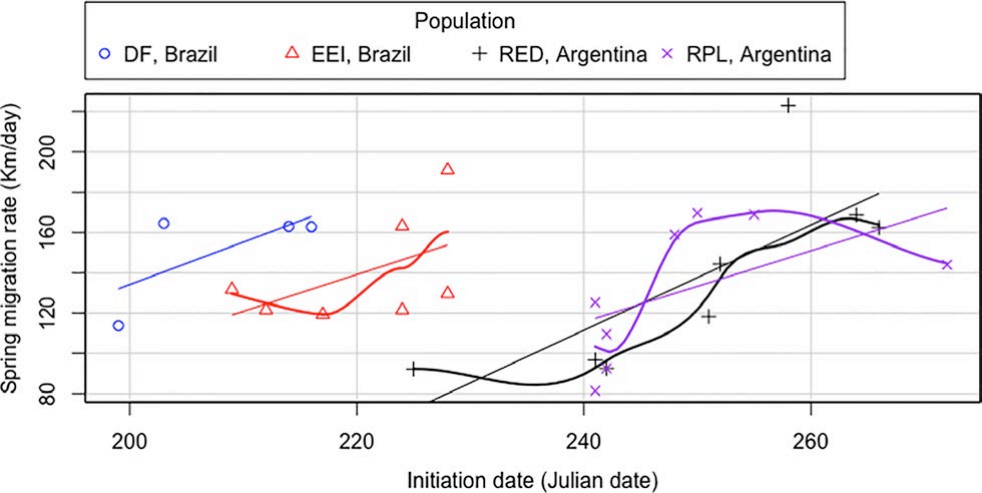
Rate of spring migration of Fork‐tailed Flycatchers as a function of date of initiation of spring migration (i.e., intratropical migrants breeding in Brazil and austral migrants breeding in Argentina). Straight lines represent the regression line for the population at each site, and curved lines represent the locally weighted smoothing line for the population at each site

There was a positive but not significant relationship between date of initiation and arrival date of spring migration for flycatchers at all sites, except EEI, Brazil (*r* = 0.81, *t = 3.12*, *df* = 5, *p* = 0.026; Figure 4), and there was a positive but not significant relationship between rate of spring migration and the date of initiation for flycatchers at all sites (Figure 5).

## 4 DISCUSSION

Overall, we found little support for the hypothesis that austral migrants are more time‐selected on spring migration than intratropical migrant conspecifics. Austral migrant flycatchers did not migrate at a significantly higher rate than conspecifics breeding at tropical latitudes, and we did not find a significantly positive relationship between date of initiation of spring migration and the rate of spring migration, a relationship that has been found in other species (e.g., Collared flycatchers in Europe, Briedis, Hahn, Krist, & Adamík, 2018). Flycatchers from across breeding sites migrated at a similar pace, regardless of their departure date, and we detected a positive but not significant relationship between departure and arrival date, a pattern which has been found in species migrating to north‐temperate breeding sites (e.g., Wood Thrush*, H. mustelina*; Stanley et al., 2012).

Research in other avian migratory systems has shown that an increase in migration distance is correlated with an increase in the rate of migration (LaSorte & Fink, 2017; La Sorte, Fink, Hochachka, DeLong, & Kelling, 2013; La Sorte, Fink, Hochachka, & Kelling, 2016). However, Fork‐tailed Flycatchers from across different sites migrated at a similar pace. Notably, the spring migration rates exhibited by austral migrant Fork‐tailed Flycatchers (i.e., ~134 km/day) are similar to those of another austral migrant flycatcher, the White‐crested Elaenia, which migrates on average between 121 and 261 km/day (Bravo et al., 2017).

Flycatchers from across all sites spent a similar amount of time on stopovers. In other migratory systems, spring migrants with larger fuel reserves have been found to depart earlier from stopover sites compared to lean birds (e.g., Goymann, Spina, Ferri, & Fusani, 2010), although a variety of factors ultimately affect an individual's timing of departure from a given stopover site (Schmaljohann & Eikenaar, 2017). Given that we have little information on the stopover ecology of either intratropical or austral migrants in South America, future research on flycatcher migration would benefit from stopover ecology research (e.g., refueling rates *en route*).

In summary, we found little evidence that austral migrant flycatchers are more time‐selected than intratropical migrant conspecifics, which could be affected by potentially high error rates associated with detecting arrival times around the spring equinox, as well as the effect of selection pressures that we did not measure, such as sex‐specific selection pressures and availability of suitable stopover habitat. Primary productivity at tropical and south‐temperate latitudes can be highly variable between years (e.g., Goetz, Prince, Small, & Gleason, 2000; Nobre et al., 2006), such that both intratropical and austral migrant Fork‐tailed Flycatchers may be under weak selective pressure to arrive as quickly as possible to the breeding grounds in any given year. Low sample sizes, especially at our tropical study sites, diminished our ability to detect differences and precluded more detailed analysis (e.g., sex‐specific patterns), such that further research on this and other species that migrate within South America is necessary to confirm our results and to test the generality of our findings.

A potentially fruitful future line of research would be to evaluate the seasonal carryover effects that Fork‐tailed Flycatchers may have to deal with when transitioning from winter to the breeding season. Fork‐tailed Flycatchers are known to track rainfall during winter (MacPherson et al., 2018), arriving at the wintering grounds in northern South America at the beginning of the wet season, which peaks in July and August (Poveda, Waylen, & Pulwarty, 2006) and where Fork‐tailed Flycatchers undergo an annual flight feather molt (Jahn, Giraldo, et al., 2016; Jahn, Seavy, et al., 2016). Winter represents a critical period during which they must properly time flight feather molt prior to spring migration, since Fork‐tailed Flycatchers generally avoid molting and migrating simultaneously (Jahn et al., 2017). Given that northern South America is susceptible to notably lower rainfall levels in some years (i.e., during the “El Niño” phase of the El Niño/La Niña climatic cycle, Poveda et al., 2006), understanding the relationship between interannual variation in food resource availability, which is key to completing feather molt (Jahn, Giraldo, et al., 2016; Jahn, Seavy, et al., 2016), and the timing of events in the flycatcher's annual cycle, will provide novel insights into the constraints molding the annual cycle and population dynamics of this and other species migrating within South America.

Further research on intratropical bird migration promises novel insights into how bird migration is molded by different environmental conditions. For example, early arrival at the breeding site by intratropical migrant Fork‐tailed Flycatchers has been shown to incur reproductive benefits (Bejarano & Jahn, 2018). Research on the extrinsic (e.g., food resource availability) and intrinsic (e.g., energetic condition) factors affecting the ability of intratropical migrant flycatchers to properly time arrival on the breeding site so as to maximize chances of successful breeding will provide important insights into the mechanisms underpinning how Fork‐tailed Flycatchers and other birds move across the tropics (Stutchbury et al., 2016).

To the best of our knowledge, this is the first comparison of individual migratory strategies between conspecific passerines breeding at tropical versus temperate latitudes. We still lack a conceptual framework on the full spectrum of ecological and evolutionary processes that shape avian migratory strategies, such as how a bird's endogenous migration program is affected by environmental conditions throughout the year (reviewed by Åkesson et al. (2017)). Thus, further comparative research on migratory strategies across different environmental contexts offers an ideal opportunity to test hypotheses regarding the constraints facing birds on migration.

The proximate mechanisms underpinning bird migration strategies are likely dependent on a dynamic interplay between intrinsic factors, such as body condition and age, and extrinsic factors, such as climate and level of competition for resources, which vary across both space and time (e.g., Bell, 2005; Guaraldo, Kelly, & Marini, 2016; Hockey, 2005). Thus, gaining a foothold on the proximate and ultimate drivers of bird migration in the tropics and south‐temperate latitudes will require a multidisciplinary, long‐term, and taxonomically broad approach. The recent advent of novel analytical techniques and miniaturized tracking technologies, such as loggers that provide combined activity and location data (Bäckman et al., 2017; Liechti et al., 2018), provides the tools necessary to employ such an approach, and ultimately shed new light on how songbirds are able to overcome the multiple challenges facing their annual spring journeys. In turn, such information will be valuable for developing effective conservation plans for migratory birds on a planet undergoing rapid habitat and climatic changes.

## CONFLICT OF INTEREST

None declared.

## AUTHOR'S CONTRIBUTIONS

A.E.J., V.R.C., D.J.L., M.A.M., M.A.P., and J.H.S. conceived the ideas and designed methodology; A.E.J., J.C., D.M., and D.T.T. collected the data; M.T.H., A.E.J., V.R.C., and D.T.T. analyzed the data, and A.E.J., V.R.C., and D.T.T. led the writing of the manuscript. All authors contributed critically to the drafts and gave final approval for publication.

## Data Availability

The data used in this study (raw light‐level data, geographic positions derived from light levels, and morphological data) are available on Movebank (movebank.org, study name: "Migratory patterns of Fork‐tailed Flycatchers (*Tyrannus s. savana*)") and are published in the Movebank Data Repository (Jahn et al., 2019).
